# Learning to never forget—time scales and specificity of long-term memory of a motor skill

**DOI:** 10.3389/fncom.2013.00111

**Published:** 2013-09-02

**Authors:** Se-Woong Park, Tjeerd M. H. Dijkstra, Dagmar Sternad

**Affiliations:** ^1^Department of Biology, Northeastern UniversityBoston, MA, USA; ^2^Institute of Computing and Information Sciences, Radboud University NijmegenNijmegen, Netherlands; ^3^Department of Biology, Electrical and Computer Engineering, and Physics, Northeastern UniversityBoston, MA, USA

**Keywords:** skill learning, retention, long-term memory, relative phase, bimanual coordination, intermanual crosstalk

## Abstract

Despite anecdotal reports that humans retain acquired motor skills for many years, if not a lifetime, long-term memory of motor skills has received little attention. While numerous neuroimaging studies showed practice-induced cortical plasticity, the behavioral correlates, *what* is retained and also *what* is forgotten, are little understood. This longitudinal case study on four subjects presents detailed kinematic analyses of humans practicing a bimanual polyrhythmic task over 2 months with retention tests after 6 months and, for two subjects, after 8 years. Results showed that individuals not only retained the task, but also reproduced their individual “style” of performance, even after 8 years. During practice, variables such as the two hands' frequency ratio and relative phase, changed at different rates, indicative of multiple time scales of neural processes. Frequency leakage across hands, reflecting intermanual crosstalk, attenuated at a significantly slower rate and was the only variable not maintained after 8 years. Complementing recent findings on neuroplasticity in gray and white matter, our study presents new behavioral evidence that highlights the multi-scale process of practice-induced changes and its remarkable persistence. Results suggest that motor memory may comprise not only higher-level task variables but also individual kinematic signatures.

## Introduction

Everybody readily asserts that people can still ride a bicycle after many years without practice. Conversely, professional pianists practice daily for many hours to maintain their high level of skill. Which aspects of a motor skill are retained and which are forgotten? What are the time scales of learning and of forgetting? Although strides have been made toward understanding declarative memory and its neural correlates in humans (Squire and Zola, 1996; Kandel, 2009; Rohrer and Pashler, 2010), long-term memory of sensorimotor skills has been largely neglected. Often regarded as a form of procedural memory, retention of sensorimotor skills is mediated by different processes and brain structures than declarative memory, as seminal studies on the patient HM have shown (Corkin, 1968; Gabrieli et al., 1993). Recent functional imaging studies on human motor skill learning and long-term retention highlighted structural changes in cortical and subcortical structures (Draganski et al., 2004; Scholz et al., 2009; Dayan and Cohen, 2011; Landi et al., 2011). An electrophysiological study on primates showed persistent changes in M1 with extensive practice after one year that was correlated with task properties (Matsuzaka et al., 2007). Neuroimaging of rodents revealed the formation and loss of dendritic spines with different amounts of practice and long-term retention (Xu et al., 2009; Yang et al., 2009). While revealing *where* and *how* neural plasticity occurs, practice-induced changes in anatomically specified locations are mute about *what* aspects of skill control are preserved and *what* behavioral correlates pertain to these plastic processes in the brain. The present study examines fine-grained changes in movement kinematics over extensive practice of a novel motor skill, retention after 6 months, and also after 8 years to assess what aspects of skill are retained and forgotten.

Studies on skill retention in humans have typically evaluated persistence across days, and very rarely longer than a few weeks (Adams, 1987; Schmidt and Lee, 2005). The amazing resurgence of skills after months or years of dormancy has only been studied in a few isolated experiments. These experiments monitored task performance in a single outcome variable, such as typing speed (Hill, 1934) and number of ball catches in juggling (Swift, 1910; Draganski et al., 2004; Scholz et al., 2009). This approach resembles methods in animal studies, where behavior is typically described by gross measures such as number of successful reaches (Xu et al., 2009) or maximum velocity achieved on an accelerating rotarod (Yang et al., 2009). Achievement of the task leaves many degrees of freedom unspecified, such as the timing of finger flexion in a reach-and-grasp action or how the rodent adjusts its running pattern to remain on the accelerating rod. This redundancy in the task allows for theoretically infinite variations in performance. What aspects are improved and retained, and which are forgotten?

To shed more light on the processes underlying the representation and formation of motor memory we performed a longitudinal study on humans practicing a novel bimanual skill and collected continuous kinematic data to afford a detailed quantitative description of the practice-induced changes. Four subjects practiced over an extensive 2-month period and were tested for retention after 6 months and 8 years. As the skill was new to all subjects, we examined learning from its inception across extensive fine-tuning and “perfecting” over 20 sessions, followed by controlled retention tests after 6 months—and in two subjects after 8 years. Detailed kinematic analyses were conducted to assess changes in kinematic behavior and their different time scales. Task-specific and task-unspecific variables were examined to assess individual styles in performance. Note that our goal was to monitor individual characteristics and their longitudinal development. We therefore focused on a few individuals in a case study fashion without any averaging across individuals.

## Materials and methods

### Participants

Seven participants were initially recruited for the experiment. From these seven only five completed the study throughout the 8 months. One participant did not show any improvements and was therefore not included in this study. The mean age of the four participants at the beginning of the experiment was 24 years, ranging between 20 and 32 years; one was female, three male. Participant 1 (male and right-handed) and Participant 2 (male and left-handed) completed the study including the 6-month retention tests. Participant 3 (male, right-handed) and Participant 4 (female, right-handed) also returned to the lab 8 years later and performed an additional retention test on the same experimental equipment. None of the participants had practiced the task before and none had formal musical training. All participants consented with the experimental procedures by signing a form approved by the Pennsylvania State University Review Board.

### Experimental apparatus

**Figure 1:**
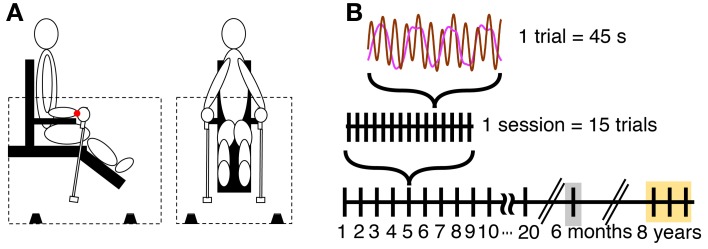
**Apparatus and task. (A)** Side and front view of the experimental setup. The dotted lines indicate enclosure, and the black objects on the floor of the enclosure indicate four microphones. The red dot at the wrist denotes the center of oscillation used for the calculation of the angular displacement of the pendulum. **(B)** Scheme of the schedule for practice and retention sessions.

Participants sat on a specially designed chair within a sound-proof enclosure (Figure 1A). Participants supported their forearms on horizontal armrests so that they could move only their wrists during the task. They grasped two identical pendulums, one in each hand, and swung them in the sagittal plane with abduction and adduction of the wrist joint. Each pendulum consisted of an aluminum rod and a wooden handle, measuring 52 cm in length. A weight of 200 g was attached at the distal end of each pendulum. To record displacements of the swung pendulums, a small sound emitter was attached at the distal tip of each pendulum that emitted low-volume beeps that were received by four microphones on the floor of the enclosure. Custom-made software computed the 3D position of the pendulums based on the travel time of sound waves from each of the emitters to the microphones. From these position data in Cartesian coordinates the software calculated angular displacements around a center of oscillation that was defined at the center of the wrist joint. This center of oscillation was defined to be at 7 cm distance from the center of the hand-held pendulum rod and 3 cm above the surface of the armrest (Kugler and Turvey, 1987; Sternad et al., 1999a). We only considered the angular displacements in the sagittal plane. The sampling rate was 90 Hz and the data were smoothed with a five-point moving average filter.

### Experimental task and design

**Figure 2:**
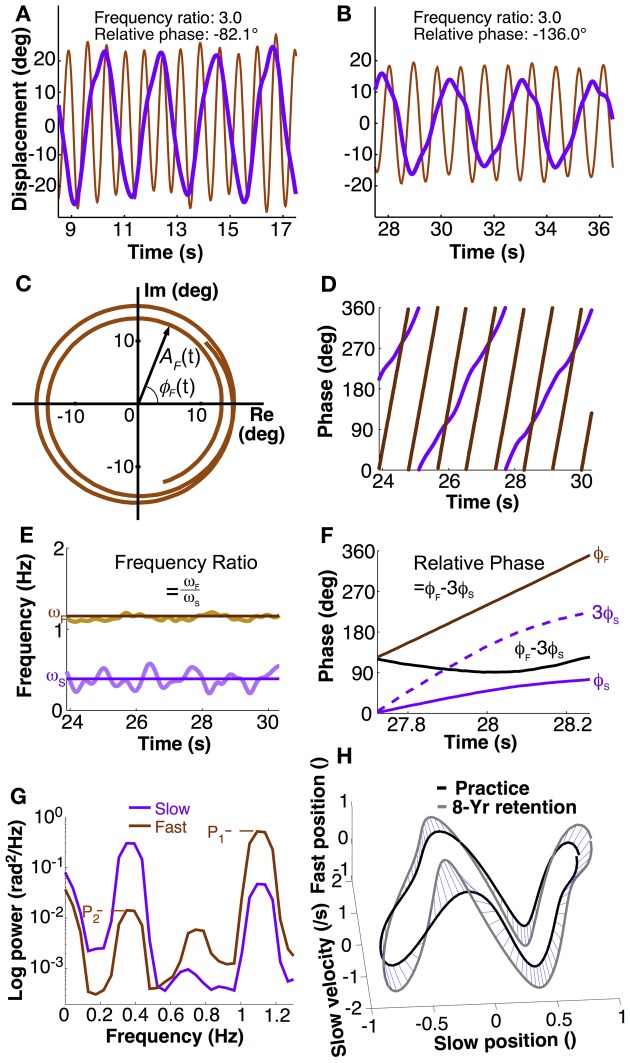
**Calculation of dependent variables. (A,B)** Exemplary trial segment showing different movement patterns of two participants. The different peak alignments indicate the different phasing of the two hands. Frequency ratio and relative phase were calculated using methods described in the text. **(C)** The Euler form of the fast hand's angular displacement, ζ_*F*_(*t*) = *A*_*F*_(*t*)*e*^*i*ϕ_*F*_(*t*)^ where A_*F*_ and ϕ_*F*_ mean the amplitude and phase in the fast hand. The time series for two and a half cycles of the fast hand angle was taken from **(B). (D)** Instantaneous phase of both hands' position calculated with Hilbert transform. **(E)** Instantaneous frequency of slow and fast hand, ω_*S*_ and ω_*F*_, calculated as time derivative of phase. Frequency ratio was the ratio of mean ω_*F*_ over ω_*S*_ (solid lines) in each trial. **(F)** Calculation of relative phase: phase of slow had ϕ_*S*_ multiplied by 3 is subtracted from phase of the fast hand ϕ_*F*_. Mean and standard deviations across one trial served as dependent measures. **(G)** Exemplary power spectral densities of fast and slow hand in a single trial to illustrate the calculation of crosstalk. Fast hand crosstalk is the ratio of the two peaks in the fast hand (P_2_/P_1_), where P_1_ is the primary peak and P_2_ is the spectral power at the movement frequency of the slow hand. **(H)** Distance between two trajectories (normalized). For visualization purpose, the distance in the 3D space is shown by blue lines between corresponding points on the two trajectories.

Participants were instructed to swing the two pendulums with a 3:1 frequency ratio such that they completed three cycles with the fast (dominant) hand while completing one cycle with the slow (non-dominant) hand. The instruction emphasized that the 3:1 frequency ratio should be achieved in a continuous fashion, unlike in drumming where movements between contacts are unspecified (Figures 2A,B). Participants were told to gaze straight ahead and swing the pendulums at their preferred frequencies and amplitudes without any further prescription. For each trial, participants were given 5 s to begin the movement before data collection was started. The experimenter demonstrated and verbally explained the task in the first two sessions. He gave qualitative verbal feedback if performance was not in line with the 3:1 frequency ratio. After having acquired the 3:1 ratio, no further instruction or feedback was provided. Note that for this rhythmic bimanual task exact error scores are not needed. As soon as performance is close to the frequency ratio, subjects converge to the desired frequency ratio, which acts like an attractor. Hence, subjects do not need explicit quantitative information about their “error”. Any small deviation would lead to non-repeating patterns, which are harder to perform. This sparse instruction also aimed to give subjects opportunity to develop their own “style” of execution. As such, this protocol emulated the everyday situation where humans learn a skill in a self-guided fashion.

The length of a single trial was 45 s, with 15 trials per session. The total movement time per session was 11.25 min and 3.75 h over 20 sessions practice. The practice period consisted of 20 sessions collected over a time of 7–9 weeks (Figure 1B). The intervals between two consecutive sessions was 2.5 days on average (40% of between-session intervals were 1 day). This was due to the varying availability of the subjects. Post-hoc tests evaluated whether different intervals between practice sessions caused different forgetting across sessions. No effect was found.

All four participants returned to the lab after 6 months to perform one retention session consisting of 20 trials. Participants performed 2 trials of the 3:1 frequency ratio, together with four other frequency ratios, 1:1, 2:1, 4:1, and 3:2 that were collected to test generalization of learning. In the interest of focus, we do not report the results of the other ratios here. In addition, we had the opportunity to collect data from two of the participants (P3 and P4) 8 years later performing the same 3:1 task in the same experimental set-up. For this test, data were collected for 3 sessions with 15 trials each on 3 consecutive days (Figure 1B).

### Dependent measures

The continuous data of the two hands were analyzed in both time and frequency domain. The dependent measures in the time domain were calculated based on Hilbert transformed data, which enabled the calculation of instantaneous estimates of phase and frequency without discontinuities due to the cyclic nature of the signal. For the calculation of the frequency-domain measure the raw displacement signals were used. Task performance was quantified by variables that were explicitly instructed as criteria for task success by the experimenter, and by variables that were unspecific to task success. For the latter measures, subjects could adopt any value and therefore express their individual “style” of performance.

### Data processing and instantaneous phase

For the calculation of the time domain measures the data were first detrended as they frequently showed a drift within a trial. This was due to the fact that amplitude and frequency were left unspecified. To eliminate this drift, a high-pass filter was applied using a 300-sample constrained least-squares FIR filter with a cut-off frequency of half the strongest frequency of the unfiltered signal. This relatively high cut-off frequency avoided confounding of the Hilbert phase estimates due to skipped cycles. To check whether the characteristics of the calculated measures during learning and retention were influenced by the high-pass filtering, we ran the same data analysis with the raw data. No qualitative differences were detected.

To obtain instantaneous phase, the angular displacements of each hand were converted to instantaneous phase using the Hilbert transform (Pikovsky et al., 2003). As any periodic function can be expressed in the complex plane, the measured displacement signal *S(t)* can be written as: ζ(*t*) = *S*(*t*)+ *iS*_*H*_(*t*), where *S*_*H*_(*t*) is the Hilbert transform of *S(t)*, and *i* is the imaginary number (Figure 2C). By Euler's formula, the periodic function is rewritten in terms of amplitude *A*(*t*) and phase ϕ(*t*) in the complex plane: ζ(*t*) = *A*(*t*)*e*^*i*ϕ(*t*)^. This equation shows that the signal consists of two separable functions of time, *A(t)* and ϕ(*t*). The instantaneous phase ϕ(*t*) can be represented in continuous or modular form between 0 and 2π, as in Figure 2D. These calculations were performed in MATLAB using the function hilbert.m (The Mathworks, Natick MA).

### Frequency ratio

The primary variable defining task success was the ratio of the frequencies of the two hands. While frequency ratio could have been computed in a cycle-by-cycle fashion, a more elegant solution was to use the instantaneous phase measure as this yielded an instantaneous frequency ratio. To begin, movement frequency of each hand was computed from the derivative of the instantaneous Hilbert phase. This phase derivative was obtained by Savitzky-Golay filtering of order 4 and length 0.5 s (Press et al., 2007). The mean instantaneous frequency over all data points within a trial represented the average movement frequency for each hand (Figure 2E). Subsequently, the ratio of the two means within one trial was obtained. The task goal was to perform with a ratio of 3.0.

### Mean and variability of relative phase

In research on 1:1 coordination relative phase has served as a measure of stability of performance. When the system is at a stable state, relative phase is invariant. Variability of this continuous relative phase quantifies the degree of stability of phase and frequency locking. As research showed, in 1:1 coordination there is a strong tendency for in-phase and anti-phase coordination. To generalize this approach to poly-rhythmic coordination, a measure should be defined that is similarly invariant when performance is stable. To account for the frequency difference and obtain an approximately constant signal, the instantaneous phase of the slow hand ϕ_*S*_(*t*) was multiplied by three and subtracted from the phase of the fast hand ϕ_*F*_(*t*) (Haken et al., 1996; Sternad et al., 1999a,b,c):

$$\phi_{3:1}(t)=\phi_{F}(t)-3\phi_{S}(t)$$

Figure 2F illustrates the calculations of relative phase between the two hands as the difference between the two phase signals. For task success, the actual relative phase could take on any value. Hence, mean relative phase is an unspecific task variable. It is an open question whether bistability, i.e. attractors at in-phase and anti-phase relations, is also observed in higher-order frequency ratios.

Successful performance implies low variability. Hence, variability of relative phase is the second task-specific variable. To quantify the mean and variability of the circular variable, the resultant vector was defined:

$$R=\frac{1}{N}\sum_{t\;=\;1}^{N}e^{i\phi_{3:1}(t)}$$

with *N* the number of samples in a trial (= 90 samples/s * 45 s). From the resultant vector the mean μ_3:1_ and circular standard deviation σ_3:1_ of relative phase ϕ_3:1_ was derived (Fisher, 1993):

$$\begin{array}{l} \mu_{3:1}=\arg(R)\\ \sigma_{3:1}=\sqrt{-2\log∥R∥} \end{array}$$

To quantify the difference of mean relative phase between the practice and retention sessions the Kullback-Leibler divergence was calculated:

$$D_{KL}(p∥q)=\int_{-\pi }^{+\pi }p(\phi_{3:1})\log\frac{p(\phi_{3:1})}{q(\phi_{3:1})}d\phi_{3:1}$$

where *p*(ϕ_3:1_) is the probability density of relative phase ϕ_3:1_ for three retention sessions, and *q*(ϕ_3:1_) the probability density of ϕ_3:1_ for the 10 last practice sessions. The probability density was obtained from histograms of trial means. Given the small number of samples, the *pdf*s were estimated using von Mises distributions fitted with the method of moments (Fisher, 1993). For convenience, the divergence measure was transformed into a similarity measure by taking the inverse:

$$Similarity=1/D_{KL}.$$

### Crosstalk between hands

Since the variables described above were derived from Hilbert phase, any changes in the movement amplitude due to the frequency “spill-over” from one hand to the other was calculated in the frequency domain. We expected that this frequency leakage may decrease over the long-term practice period, reflecting increasing inhibition of intermanual crosstalk. As we did not prescribe the amplitude of the oscillatory movements, the crosstalk measure was a task-unspecific variables.

For each hand's angular displacements, the power spectrum for each trial was obtained by applying the Welch procedure with 3 segments and 50% overlap between the segments (Press et al., 2007). The amount of crosstalk across hands was quantified by the ratio of the primary and secondary peaks in each hand's spectrum (Figure 2G). Each hand's power spectrum showed a minimum of two pronounced peaks: the primary peak, pertaining to the main oscillation frequency, and a secondary peak that potentially reflected the influence of the other hand's frequency. However, in the slow hand, higher harmonics potentially coincided with the frequency of the fast hand. Therefore, only the fast hand spectrum was examined to quantify crosstalk (Figure 2G). While the primary peak (P_1_) was unambiguous, the secondary peak (P_2_) was identified by first determining the slow hand's primary frequency and then selecting the highest power within a window of ±10% of the peak frequency of the slow hand. The ratio of the two peak powers (P_2_/P_1_) served as a measure of the crosstalk.

### Trajectory similarity

A final variable was defined to quantify the similarity of the continuous trajectories in 4-dimensional state space across practice and retention. This similarity measure was used to quantify longitudinal changes within each individual, specifically how much was retained from the end of practice to the retention sessions.

Adopting a dynamic interpretation of the hands' trajectories, the state space of each hand is defined by its angular displacement and angular velocity. Hence, the state space of the bimanual movements is four-dimensional. In order to assess whether the bimanual coordination pattern preserved its individual characteristics across the two long-term retention intervals, a distance measure was developed in four-dimensional state space to compare two trajectories. Given that state space does not have a metric because position and velocity have different units, normalization of position and velocity in both time and amplitude was necessary. Starting with the time series, we normalized the amplitude by setting the slow amplitude to one and scaling the fast amplitude accordingly. The amplitude-normalized trajectories were differentiated with a Savitzky-Golay filter of order 4 and a window size of 0.5 s to obtain angular velocity. Next, we calculated an average trajectory by parsing angular displacement trajectories into cycle segments, with one segment defined as spanning one slow cycle. Both slow and fast hands' trajectories were parsed at peak extension of the slow hand and time-normalized by interpolating the time series to a new time grid with 100 points. An average cycle was then calculated over all cycles within a session. With this normalization the distance between the two trajectories could be calculated. The pairwise distance *d* was computed in a point-wise fashion:

$$d_{i}=\sqrt{\sum_{j\;=\;1}^{4}{\left(p_{ij}-q_{ij}\right)}^{2}}\text{ and }d=\frac{1}{100}\sum_{i\;=\;1}^{100}d_{i}$$

with *p*_*ij*_ and *q*_*ij*_ denoting two orbits, *j* denotes the *j*th coordinate (N_*j*_ = 4) at the *i*th time (N_*i*_ = 100). As the purpose of the comparison was to compare practice performance with retention performance, the last practice session was compared with the last retention session. For convenience, the distance measure was transformed into a similarity measure by taking the inverse. A 3D version of these calculations is illustrated in Figure 2H. Note that this measure does not create a true or meaningful metric. However, visual checking showed that the measures scaled very well with what was seen by eye.

### Statistical analyses

To assess the time scales of change in the different variables, the time course of variability of relative phase was fitted by an exponential curve to obtain time constants. For intermanual crosstalk, a linear fit was used because the variable was obtained from the power spectrum and hence was a logarithmic quantity.

The main experimental hypothesis was that subjects would forget, i.e., there was a difference between the last practice session and the first retention session after 6 months and 8 years. Statistical comparisons were conducted within individuals, not across individuals, as statistical power for four subjects was insufficient. As such, this study is a case series. To evaluate the differences between practice and retention sessions, unpaired Mann–Whitney-*U* tests (Wilcoxon rank sum test) compared several movement variables in the 15 trials of the last practice session with the 2 trials collected after 6 months. We chose non-parametric tests as the 6-month session only had 2 trials and the practice data failed to show normal distributions, as tested by the one-sample Kolmogorov-Smirnov test. The same Mann–Whitney-*U* tests were performed on the last 15 practice trials with the first 15 trials collected immediately after 8 years. Trajectory similarity was evaluated between the last practice trials, the 6-month retention trials and the third/last session of the 8-year retention trials. This comparison tested the stable performance style excluding the short familiarization period. For all statistical comparisons *p*-values of <0.05 were deemed significant. All analyses were performed in MATLAB (The Mathworks, Natick, MA).

## Results

The results describing the time course of learning and its long-term retention are grouped according to the task variables, starting with task-specific variables to assess whether subjects acquired the skill, followed by task-unspecific variables to describe each individual's time course of learning and retention. For each variable, we first present its learning characteristics followed by assessment of its retention.

### Frequency ratio and variability of relative phase

**Figure 3:**
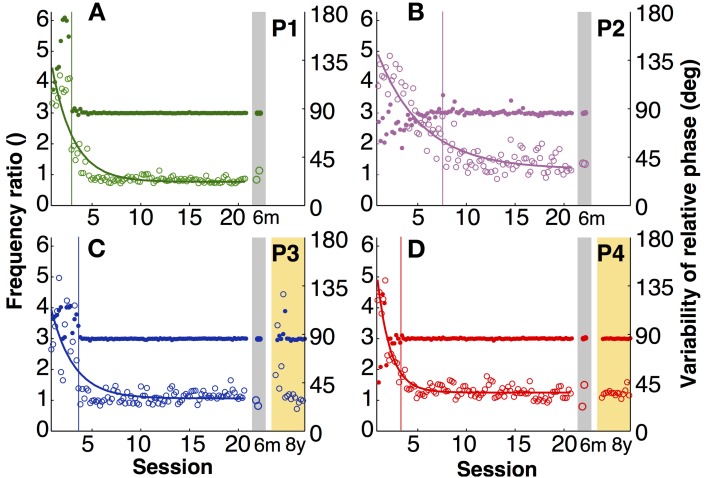
**Task-specific variables. (A–D)** Frequency ratio (filled) and variability of relative phase (open) for each participant across 20 practice sessions and the 6-month (gray shade) and 8-year retention tests (yellow shade). Each data point is the average across three trials (5 points per session). The thin vertical lines indicate where the standard deviation of the frequency ratio in a moving window of 15 successive trials was 3 times greater than that of the last 10 sessions (moving from late to early trials).

The explicit instruction to the participants was to produce a frequency ratio of 3:1, with as little variability as possible. Note that task instructions did not require a specific absolute frequency, nor a specific amplitude or relative phase. To assess whether participants accomplished this task, frequency ratio and variability of relative phase were analyzed. Figures 3A–D shows time series of the four participants of both variables. To compress the graphic representation of relative phase and its variability, the means of 3 consecutive trials were pooled with no overlap (5 data points per session, 100 data points during practice). Given the small amount of data in the 6-month retention session, the two trials were represented separately.

The four participants acquired the desired frequency ratio after 3, 7, 4, and 3 sessions, respectively. After some initial variability, they all “discovered” the correct frequency ratio rather abruptly and then never lost this pattern again. This contrasts with the time course of variability of relative phase, which changed more slowly and smoothly over the course of practice. The decline was approximately exponential reaching a plateau after 5–7 sessions. Exponential fits with a single time constant quantified the change in this variable, showing values between 1.4 and 5 (in units of sessions) for the four participants (Table 1).

**Table 1:** **Time constants (τ) of change in mean and variability of relative phase and intermanual crosstalk**.

**Participants**	**Time constant variability of relative phase**	**R**^**2**^	**Time constant mean relative phase**	**R**^**2**^	**Time constant intermanual crosstalk**	**R**^**2**^
P1	2.2 [1.9–2.8]	0.82	N/A	N/A	6.0 [5.0–7.2]	0.55
P2	4.7 [3.9–5.0]	0.84	5.5 [3.5–12.3]	0.49	6.2 [5.3–7.6]	0.54
P3	2.2 [1.7–3.5]	0.60	4.0 [2.3–14.7]	0.29	7.0 [6.0–8.3]	0.62
P4	1.4 [1.2–1.6]	0.85	N/A	N/A	6.7 [5.9–7.8]	0.67

Time constants for variability and mean were obtained from exponential fits to the variables over the 20 practice sessions: $y(t)=y_{0}e^{-\frac{t}{\tau }}+y_{\infty }$. Time constants for crosstalk were the slope parameters of the linear fits of the log-transformed values: $log\left[y(t)\right]=C-\frac{t}{\tau }$. Values are in units of session; values in parentheses denote the 95% confidence interval of each fit.

Having ensured that all four participants acquired the skill, retention could be tested. The last 15 trials of practice session 20 were compared with the two retention trials of the 6-month tests using unpaired Mann–Whitney-*U* tests. Frequency ratio revealed no significant differences (*p* > 0.1), indicating that none of the four participants showed significant forgetting after 6 months. For the two participants that repeated the experimental task again after 8 years, their 8-year performance was compared with their last practice session and also with their 6-month retention. The frequency ratio did not show any significant changes (*p* > 0.1), indicating no signs of forgetting in their ability to achieve the task.

Variability of relative phase similarly remained low after 6 months. None of the four participants revealed significant differences from the final 15 trials of practice (*p* > 0.1). After 8 years one participant's (P4) variability was statistically unchanged. However, P3's variability in the first retention session increased slightly (*p* < 0.01; Figures 3C,D). Nevertheless, this test lost significance (*p* > 0.05) when the second and third session of the 8-year tests were used for comparison, showing that there was rapid relearning.

### Mean relative phase

**Figure 4:**
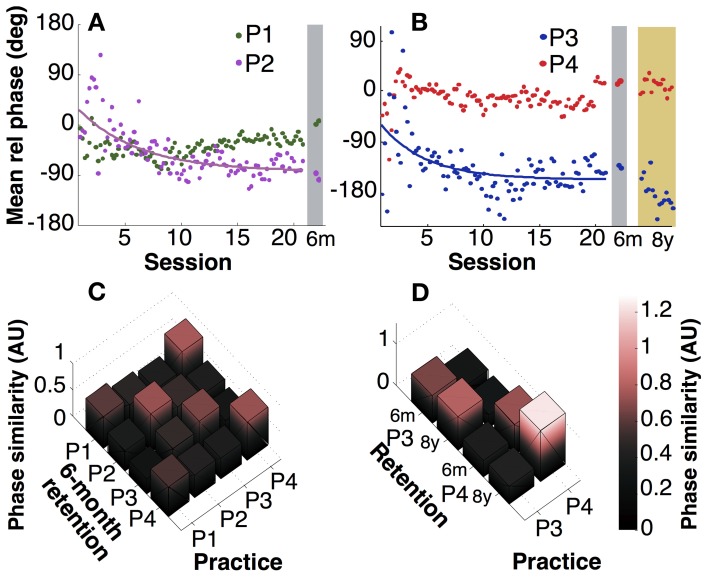
**Mean relative phase. (A,B)** Mean relative phase of participants 1 and 2 across practice and at 6-month retention test and participants 3 and 4 including 8 years. **(C)** Pairwise comparison of individuals' relative phase between practice (last 10 sessions) and 6 months retention. AU: arbitrary unit. **(D)** Pairwise comparisons between 6-month and 8-year retention performance.

The instructions were kept deliberately sparse to allow for individual preferences or styles. A first look at exemplary time profiles of the four participants showed that they indeed adopted different styles of execution, marked by different relative phases, frequencies, and also slightly different waveforms, while achieving the desired 3:1 frequency ratio (Figures 2A,B). Motivated by this observation, we analyzed mean relative phase. Figures 4A,B shows that participants developed different relative phase patterns across practice: P1 and P4 exhibited relatively consistent mean relative phase throughout practice (−24° and 21°), while P2 and P3 converged toward −75° and −140°, after initial fluctuations. For the latter two participants, the gradual change of the mean relative phase was fitted with exponential curves. The time constants were 5.5 and 4.0 sessions, respectively (see Table 1). These time scales were longer than those of variability of relative phase.

Looking at retention, mean relative phase did not change significantly after 6 months (*p* > 0.1), except for P1, who exhibited a small change (*p* = 0.02, Figure 4A). However, it is also clear from comparisons across participants that their individually preferred phase values were largely preserved. To highlight this individual persistence, a similarity metric (inverse of Kullback-Leibler divergence) quantified the differences between practice and retention within and across participants (Figures 4C,D). Figure 4C summarizes the pairwise comparisons of four participants between practice and 6-month retention. With one exception (P1 compared to P4), the highest values are on the diagonal, signaling that retention performance was more similar to practice performance of the same individual compared to other individuals. The comparison between the practice sessions and the three 8-year retention sessions in Figure 4D also showed persistence. However, pairwise statistical comparisons of trial means for P3 and P4 detected small but significant differences (*p* < 0.05). Even though statistically significant, it is also obvious from the panels A and B that the differences between participants in mean relative phase were larger than the differences within an individual. It should be emphasized again that the relative phasing between the two hands was completely unconstrained, and thus represented the “style” that each person adopted.

### Movement frequency

**Figure 5:**
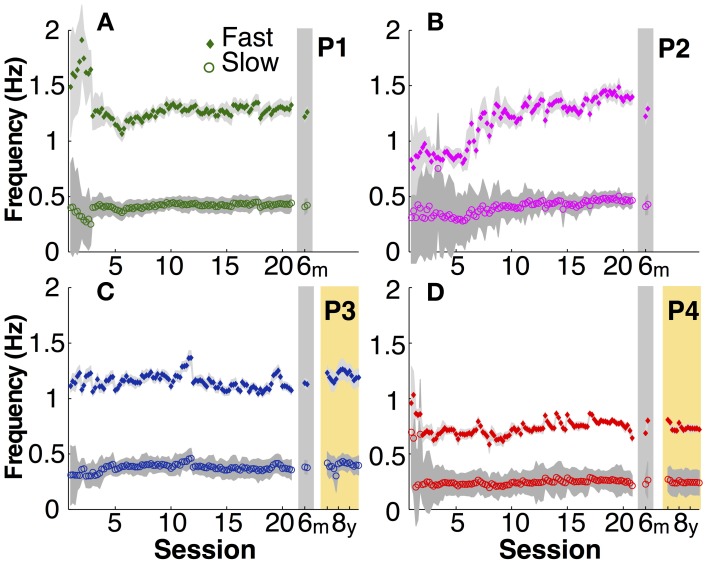
**Actual movement frequency. (A–D)** Mean fast (filled diamond) and slow (open circle) hand movement frequency in each participant (P1 to P4) over practice and retention. Gray shadows indicate one standard deviation.

Similarly unspecified in the instructions were the actual movement frequencies that the two hands adopted. As Figure 5 illustrates, after initial variability particularly in the fast hand of P1 and P2, the movement frequencies in both hands became fairly constant in the second half of the practice sessions. Comparison between participants showed that the adopted frequencies in the slow hand ranged between 0.2 and 0.4 Hz and the fast hand adopted three times this frequency. The fast right hand showed markedly higher variability in early practice, but then achieved the 3:1 frequency locking in a stable manner.

In the 6-month retention trials, the slow hand frequencies remained within the 95% confidence interval of the second half of the practice sessions. In the 8-year retention trials, the slow hand frequencies were within the 95% confidence interval of the last 10 practice sessions in both participants. The same persistence was observed in the fast hand frequencies.

### Intermanual crosstalk

**Figure 6:**
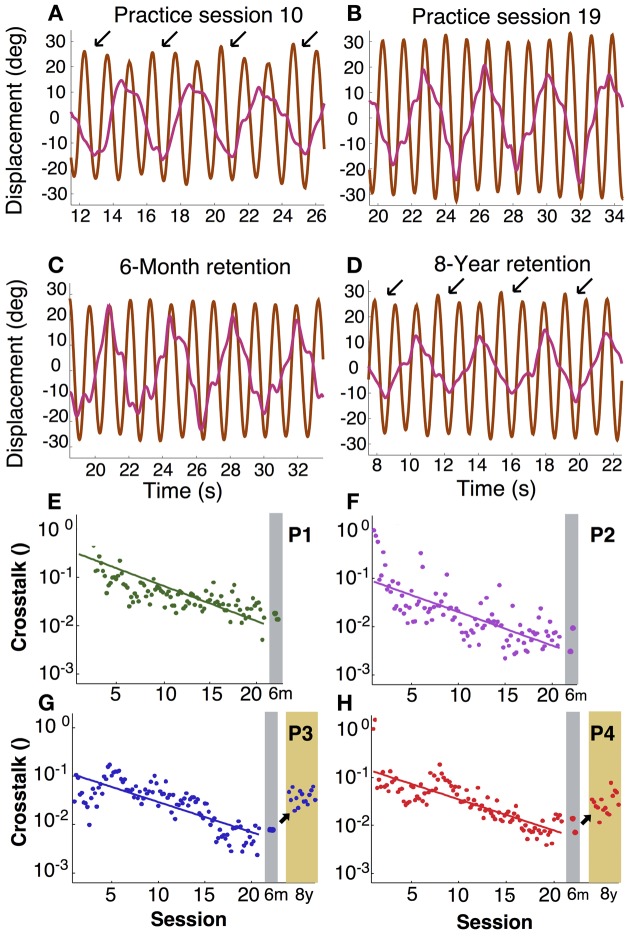
**Intermanual crosstalk. (A–D)** Exemplary angular displacement profiles in the fast (brown) and slow (pink) hand of participant 4. In the middle of practice **(A)**, accentuated peaks at every three cycles (arrows) were observed. At the end of practice and 6-month retention, accentuation disappeared **(B,C)**. In an 8-year retention trial, the accentuated peaks (arrows) appeared again **(D)**. **(E–H)** Learning and retention of intermanual crosstalk in each participant (P1 to P4). Crosstalk was log-transformed and fitted with a linear function: $\log\left[y(t)\right]=C-\frac{t}{\tau }$ The inverse slope parameter equals the time constant of change.

In asymmetric bimanual tasks interference across the two hands can be expected. This is exemplified in the amplitudes of the displacement profiles. Inspecting the unfiltered profiles, the fast hand showed that the amplitude of every third cycle was higher than others, especially during early practice (Figure 6A). This accentuation caused by the slow hand was reduced during late practice, suggesting fine-tuning due to learning. This modulation remained low at the 6-month retention test, but recurred after 8 years (Figures 6B–D). This periodic infiltration of the slow hand led to an increase in spectral power in the fast hand frequency and was quantified in the crosstalk measure. Figures 6E–H shows in four participants how this measure continuously declined during practice. Note that the crosstalk measure is a logarithmic quantity. Therefore, the time course of the crosstalk was fitted with a linear regression. The time constants or slopes of the linear fits were 3–5 times longer than the time scales for the variability of relative phase in the same individual (except for P2, Table 1).

After 6 months of retention, the values remained low at the practice level; Mann–Whitney-*U* tests did not reveal differences for P1 to P4 (*p* > 0.1, *p* > 0.7, *p* > 0.5, *p* > 0.4, respectively). However, after 8 more years, the two participants showed significant increases (*p* < 10^−9^ for both). Importantly, there were also no signs of relearning as observed for variability of relative phase.

### Trajectory similarity between practice and retention

**Figure 7:**
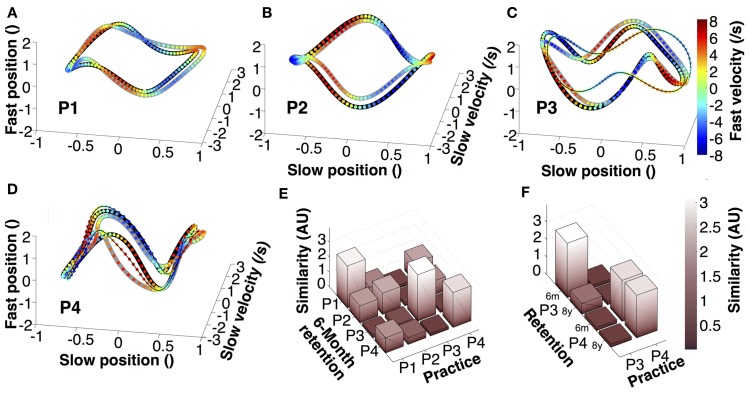
**Average trajectories in four-dimensional state space. (A–D)** Representation of average trajectories for each participant during the last practice session (thick black lines), 6-month retention (thick gray lines), and 8-year retention session (thin black lines). **(E,F)** Similarity between the trials of the last session, the 6-month retention trials and the 8-year retention trials of participants 3 and 4.

To further test the retention of individual movement characteristics, we examined the continuous trajectories of both hands in state space (Figure 7). Position and velocity of individual cycles of the slow and fast hands were averaged, normalized, and displayed in 4-dimensional state space (fourth dimension is color). Figures 7A–D presents the orbits of the four participants, each panel showing one averaged practice trial, one 6-month retention trial, and, for P3 and P4, an 8-year retention trial. The different 4D patterns were generated by the relative phase and amplitude ratio between the two hands in the four participants. These graphs illustrate that within an individual there was little qualitative change in the continuous kinematics. A similarity measure, computed from the pairwise distances between the orbits in normalized state space, quantified the deviations between two orbits. Figure 7E summarizes the differences between all four participants in practice and 6-month retention in matrix format. Comparing 6-month retention with practice, each individual's 4D-trajectory was closest to the trajectories of the same individual, shown by the highest values in the diagonal (even though the result of P2 is less strong and its magnitude is comparable to three other pairwise comparisons). Figure 7F summarizes the results between practice and retention for P3 and P4. Again, the participants' similarity measures were visibly higher when comparing within themselves.

## Discussion

Using an asymmetric bimanual skill that comprised *de novo* learning and extensive fine-tuning or “perfecting,” the longitudinal case study on four subjects presents a detailed characterization of learning and retention. We defined multiple kinematic measures that reflect not only task achievement but also individual styles of performance. The statistical analyses were performed within each individual without averaging across individuals or group comparisons. This approach is consistent with the growing understanding that averaging may mask characteristic time scales within individuals, especially in more complex skills where they can develop markedly different styles (Newell et al., 2001; Cesqui et al., 2012). Further, numerous imaging studies reported significant inter-individual differences, suggesting that group analyses may obscure results on motor skill learning (Schlaug et al., 1994; Pascual-Leone et al., 1996; Müller-Dahlhaus et al., 2008). It is all the more noteworthy that at the backdrop of idiosyncratic differences in “style” variables, the four individuals also showed a remarkably similar pattern of results in task specific variables.

The results reveal that different kinematic features show different time scales of change during the two-month-long practice period and across the long retention interval. Despite 6 months and 8 years of dormancy, all but one measure of skill showed remarkable persistence, regardless of their specificity to the task. Our behavioral results point to the multiplicity of neuroplastic processes and the specificity of motor memory. In the following we proceed chronologically and first discuss the results on learning, collecting the findings from all variables, before proceeding to retention, as skill acquisition is the necessary precursor to retention.

### Time scales of learning

Our results show that in all four subjects task-specific and unspecific variables show different temporal profiles of change over the extensive practice period. The primary task criterion, frequency ratio, was acquired after some searching over 3–5 sessions in an almost discontinuous “discovery” fashion. It is noteworthy that none of the participants ever deviated from this task-specified ratio again. Acquiring a rhythmic coordination pattern proceeds differently from learning a discrete pointing task. The error is not a continuous variable that requires precise feedback to be reduced. Rather, the task-required frequency ratio is a solution that has attractor properties, once the performance is within the basin of attraction. While the present study did not explicitly probe the attractor property by applying perturbations, a number of previous observations are consistent with this interpretation (Kay et al., 1987, 1991). Numerous studies on rhythmic bimanual coordination showed that subjects immediately lock into a 1:1 frequency ratio and deviations from exact in- or anti-phase relationship are only induced when the two hands have different natural frequencies. These features of bimanual rhythmic coordination have been successfully modeled by coupled oscillator models (Sternad et al., 1992, 1995; Treffner and Turvey, 1995). Stated in more subjective terms, subjects avoid deviations from invariant phase relations as this implies non-repeating patterns that require effort or attention. The same observation holds for slightly more demanding frequency ratios, such as 1:2 or 2:3 (Peper et al., 1995; Sternad et al., 1999b,c).

In contrast, other variables exhibited more gradual, approximately exponential changes, albeit with different time constants. The measure of intermanual interference, the only variable calculated in the frequency domain, decreased at the slowest rate. Note that the variability measure was different across individuals while the crosstalk measure was comparatively similar across individuals. The slow change gives expression to the continuous, seemingly never-ending “fine-tuning” of the skill: note that the frequently cited data on increasing cigar rolling speed with many years of practice is a cross-sectional study and the data were obtained from different individuals (Crossman, 1959). Interestingly, this measure of fine-tuning was also the one with least persistence, consistent with the anecdotal observation that the finer aspects of a skill, such as in artistic piano playing are forgotten relatively quickly, while the gross motor skill is maintained.

One reason why several different time scales could become apparent was that, unlike in studies on error-based learning of highly controlled tasks, the instruction left many performance aspects free to choose, such as the individual hands' frequency, amplitude, and relative phase. Participants had the opportunity to develop and stabilize their preferred realization of this 3:1 ratio in a largely self-driven fashion. As such, our experiment revealed self-guided tuning of a skill toward an individually preferred stable pattern.

One task-unspecific variable was relative phase between the two hands' cycles. It was not specified by the task and yet, each individual established their own preferred value. Note that it is unlikely that biomechanical constraints determined the individually chosen relative phases. Individuals converged to very different values that spanned the entire range, even though the anatomy of the wrist can be assumed to be sufficiently similar. Two participants started with close to in-phase coordination but then diverged, one to arrive at a relative phase close to 90 degrees, while the other asymptoted to 180 degrees. This spectrum of preferred relative phases differed from findings in studies on 1:1 bimanual coordination where humans invariably adopted only two states, in-phase and anti-phase (Haken et al., 1985; Sternad et al., 1992, 1999a). This behavior has been interpreted as stable attractor states, because coupled oscillator models provided a good account of rhythmic bimanual coordination. The observed convergence toward a preferred pattern within two individuals in this study may be a hint that an attractor develops with practice.

The tenet that skill acquisition involves a multiplicity of processes with different time scales is not new (Newell et al., 2001; Kiebel et al., 2008). However, most behavioral studies on motor learning and adaptation relied on descriptions with a single variable, typically an error variable, which makes it harder to reveal this multifaceted process. The debate on single or multiple underlying processes was grounded in whether exponential curves or power law curves were the more appropriate model fits for learning curves (Newell and Rosenbloom, 1981; Heathcote and Brown, 2000). Exponential fits express a single time constant while power laws incorporate many time constants. Explicit tribute to the existence of two time scales is found in modeling work by Smith and colleagues (Smith et al., 2006). Studying adaptation to externally applied force fields in a reaching task, the iterative learning model includes the superposition of two component processes with different time constants (Joiner and Smith, 2008; Sing and Smith, 2010). While this elegantly simple model successfully captured a variety of learning and forgetting characteristics, the experimental task is one of adaptation or compensation, not *de novo* learning, which arguably relies on different processes in plasticity.

Evidence for slow and fast processes are highlighted in several neuroimaging studies. Use-dependent changes in gray matter have been documented in a network of motor cortex, cerebellum, and posterior parietal cortex, reflecting plastic processes with fast turn-over, such as synaptogenesis and dendritic arborization (Landi et al., 2011). Slower-evolving mechanisms, such as neuronal or glial cell genesis, have been shown in animal studies (Kleim et al., 2007) and changes in white matter, such as myelination or packing density of nerve fibers, indicated by changes in fractional anisotropy, have been documented in two human studies on juggling (Draganski et al., 2004; Scholz et al., 2009). An fMRI study on learning bimanual coordination showed decreasing activity in the fronto-parietal region and increasing activity in the motor cortex and basal ganglia as practice progressed, suggesting that different cortico-subcortical pathways are activated during early and late practice (Debaere et al., 2004). However, these imaging studies did not establish correlations to overt performance quality. Our study adds behavioral support for this multiplicity of neuroplastic processes by examining continuous kinematic data documenting both task-specific and unspecific aspects.

### Long-term retention and its specificity

Studies on skill retention in humans have typically evaluated persistence of a learnt skill across hours and days, and rarely longer than a few weeks (Adams, 1987; Schmidt and Lee, 2005). A few isolated experiments reported remarkable long-term retention, but these studies used relatively coarse outcome variables, e.g., typing speed and number of ball catches in juggling (Swift, 1910; Hill, 1934; Draganski et al., 2004; Scholz et al., 2009). Similarly, behavioral descriptions of performance in animal studies on neuroplasticity do not match the astonishingly detailed imaging of single-neuron changes. Dendritic spine counts in specified neurons is associated with percent successful reaches or maximum velocity on an accelerating rotarod (Xu et al., 2009; Yang et al., 2009). To the best of our knowledge, the present study is the first to quantify finer-grained kinematic measures over months and years. Needless to say, it would be desirable to have both detailed behavioral and neuronal documentation.

Our results document that after six months, and in two cases also after 8 years, participants reproduced not only task-specific but also task-unspecific features of the skill with little or no re-learning. Within-participant comparisons of the continuous movement trajectory in 4D phase space showed that each participant retained the overall topological features of their performance. While the measure was performed on normalized trajectories and cannot be interpreted in absolute terms, the similarity within individuals contrasted visibly with the differences between individuals. The relative invariance of trajectories within an individual is reminiscent of what has been observed in handwriting: everybody has his/her own idiosyncratic signature that is preserved across time and even across limbs, at least qualitatively (Bernstein, 1967; Raibert, 1977). However, no explicit quantification has been attempted of what is preserved and what is forgotten. Further, this skill is usually continuously practiced and therefore not a good test of long-term persistence over a period of no practice.

One exception to the long-term persistence of skill features is the frequency leakage across hands, indicating that attenuation of intermanual crosstalk may have less long-term stability. Note though, that the 6-month retention tests showed low values, and the crosstalk measure only returned to initial values after 8 years. One explanation for this differential time course is that the crosstalk measure was calculated from displacement data that included both phase and amplitude, in contrast to the computation of relative phase and frequency that only considered phase (see Figure 2C). This may suggest that kinematic aspects including amplitude information were less stable and may reflect distinct neural specifications.

Bimanual synchronized performance is mediated by the corpus callosum, as evident in acallosal patients who are less able to maintain continuous phase-locking in a bimanual task (Franz et al., 1996; Kennerley et al., 2002; Sternad et al., 2007). A recent neuroimaging study on healthy humans also verified that skilled bimanual performance is correlated with the degree of white matter integrity of the corpus callosum (Johansen-Berg et al., 2007). Similarly, extensive piano practicing induces plasticity in the white matter of the corpus callosum, especially during maturation in childhood (Bengtsson et al., 2005). However, it is yet an open question whether changes in white matter are possible beyond the maturation stage. An intriguing question is whether crosstalk is an indicator of interhemispheric connections and whether the slow times scales reflect white matter changes due to the extensive practice. However, a more conclusive answer to this question will require neuroimaging.

Although the present study is purely behavioral, the long-term persistence of idiosyncratic features invites some speculations about what the brain encodes. Previous work on primates (n = 2) using single-neuron recordings showed that after extensive practice of a sequencing task neural activation changes in M1 correlated with task properties (Matsuzaka et al., 2007). Behavioral research on a sequential reaching task in humans also revealed differential interference and consolidation between an explicit task success indicator (learning the correct sequence) and an implicit performance measure (spatial accuracy), consistent with the distinction between task vs. movement error learning (Ghilardi et al., 2009). Our results suggest that even finer-grained kinematic specifics generated by muscular activation sequences may be encoded in neural networks in a long-term stable fashion. This interpretation differs from the view that control of sensorimotor actions is structured hierarchically, with representation of higher-level task goals that recruit subordinate individual realizations that are never exactly the same (Bernstein, 1967; d'Avella et al., 2003). Even though the observed specificity of motor memory may be due to the extensive and monotonous practice in our study (not unlike in primate studies and other highly controlled experimental tasks), our results may still add to the discussion on what is represented in the brain (Ebner et al., 2009).

### *De novo* learning vs. adaptation

A final remark on our novel experimental approach and the chosen model task is in order as it contrasts to much current research on adaptation of a discrete reach to externally imposed perturbations, such as force fields or visuomotor rotations (Shadmehr and Brashers-Krug, 1997; Krakauer et al., 2000; Wu and Smith, 2013). Our task and learning paradigm differs in numerous aspects: To begin, our focus was on *de novo* learning, i.e., acquiring a motor skill that is not part of the archetypal human repertoire, such as reaching, walking, or grasping. While beyond the functionally necessary, learning new challenging skills appears to be a specifically human desire: seemingly “useless” skills, such as skateboarding, performing a back-flip, or manipulating a Rubik's cube, seem to have universal appeal. The body of research on motor adaptation examines how a previous core behavior, a reach with the largely invariant straight line and bell-shaped velocity profile, is *re*-established during or after perturbation. It is tempting to speculate that these two scenarios of performance changes are mediated by different underlying neural processes.

We chose a rhythmic bimanual skill that presents moderate complexity but is still achievable by all individuals. Performing the 3:1 frequency ratio requires sufficiently long acquisition time, or “dynamic range,” to allow multiple time scales to be observed in different performance variables. The rhythmic bimanual skill also affords quantification by a variety of dependent measures, beyond the common error measures, that were inspired by previous modeling work with coupled oscillators (Haken et al., 1996; Sternad et al., 1996, 1999c). Extending from this line of work, it would be interesting to model learning and retention with similar coupled oscillator models.

One other potentially important difference from reach adaptation studies is that our chosen model skill is rhythmic. To begin, our rhythmic task that does not have any visual model and, hence, no visual error, unlike target-directed reaching that is under significant visual control (Shabbott and Sainburg, 2009). Further, in previous behavioral and imaging work, we have presented evidence that discrete and rhythmic movements may be under different neural control (Sternad et al., 2000; Schaal et al., 2004). We and others have argued that these two types of actions constitute two different primitives (Schaal et al., 2000; Hogan and Sternad, 2007, 2012; Ispeert et al., 2013). Two recent behavioral studies using the adaptation paradigm have reinforced this distinction: Ikegami et al. (2010) showed that adaptation to altered visuomotor conditions was almost fully transferred from discrete to rhythmic performance, while there was minimal transfer in the reverse direction. Howard et al. (2011) reported that when learning reaching movements in force fields with different directions, interference between reaching in different force fields was reduced when each field was performed in either a rhythmic or discrete manner. Aside from these and some studies on juggling and bouncing a ball, there has been little research on learning a rhythmic skill (Beek and Turvey, 1992; Sternad, 1999; Dijkstra et al., 2004; Wei et al., 2008). More work on learning rhythmic skills is needed.

Lastly, when acquiring a novel skill, humans invariably develop their idiosyncratic styles, partly because quantitative error feedback is commonly absent. This real-world scenario differs from the usual strictly controlled experimental paradigms where precise quantitative feedback is made available, sometimes even in a time-controlled manner. Our study deliberately refrained from specifying explicit target behaviors and providing error information, allowing for individual preferences to surface. While this self-guided learning leads to considerably more variability that may present a challenge to the experimenter and theorist, it does allow intrinsic tendencies to become apparent. That the observed individual differences are not random, changing with day and mood, but rather get established and engrained with practice, is supported by our retention results. Seemingly fleeting style features are preserved over 6 months and also over 8 years! Consistent with several voices in the research community, individual performance has been a neglected domain and needs more attention in motor learning studies (Kandel, 2000).

In conclusion, we showed that behavioral measures sensitively reveal multiple time scales, suggestive of the multiplicity of parallel neuroplastic processes in the central nervous system. Our data also provided quantitative evidence for the long-term stability of specific skill characteristics—in two subjects even after 8 years. In contrast, the resurgence of intermanual crosstalk in 8-year retention suggests that these processes may have different persistence and may underlie the observed partial loss of skill without continued practice, as for example the loss of “fluency” in piano players. Combining such detailed behavioral quantification with state-of-the-art neuroimaging may reveal connections between neural processes and its behavioral correlates, important knowledge for theoretical development and practical applications in rehabilitation.

### Conflict of interest statement

The authors declare that the research was conducted in the absence of any commercial or financial relationships that could be construed as a potential conflict of interest.
